# Active pulmonary tuberculosis: something old, something new, something borrowed, something blue

**DOI:** 10.1186/s13244-021-01138-8

**Published:** 2022-01-09

**Authors:** Maria T. A. Wetscherek, Timothy J. Sadler, Janice Y. J. Lee, Sumit Karia, Judith L. Babar

**Affiliations:** 1 Department of Radiology, Addenbrooke’s Hospital, Cambridge University Hospitals NHS Foundation Trust, Hills Rd, Cambridge, CB2 0QQ UK; 2 Department of Pneumology, Iuliu Hatieganu University of Medicine and Pharmacy, 8 Victor Babeș Street, 400000 Cluj-Napoca, Romania

**Keywords:** Pulmonary tuberculosis, X-ray, Computed tomography, Imaging, Immunocompromised

## Abstract

Tuberculosis remains a major global health issue affecting all countries and age groups. Radiology plays a crucial role in the diagnosis and management of pulmonary tuberculosis (PTB). This review aims to improve understanding and diagnostic value of imaging in PTB. We present the old, well-established findings ranging from primary TB to the common appearances of post-primary TB, including dissemination with tree-in-bud nodularity, haematogenous dissemination with miliary nodules and lymphatic dissemination. We discuss new concepts in active PTB with special focus on imaging findings in immunocompromised individuals. We illustrate PTB appearances borrowed from other diseases in which the signs were initially described: the reversed halo sign, the galaxy sign and the cluster sign. There are several radiological signs that have been shown to correlate with positive or negative sputum smears, and radiologists should be aware of these signs as they play an important role in guiding the need for isolation and empirical anti-tuberculous therapy.

**Supplementary Information:**

The online version contains supplementary material available at 10.1186/s13244-021-01138-8.

## Key points

- Radiology plays a crucial role in the diagnosis of active pulmonary tuberculosis (PTB).
- Imaging appearances of PTB depend on immune status.
- Radiological findings of PTB may mimic other diseases.
- CT findings correlate with sputum smear positivity.

## Background

According to the World Health Organisation (WHO), tuberculosis (TB) remains in the top 10 causes of death worldwide with approximately 10 million cases diagnosed in 2019. TB is present in all countries and age groups. Substantial efforts have been made as part of the WHO End TB strategy with a resulting 9% decrease in global incidence between 2015 and 2019. An estimated 60 million lives have been saved through improved diagnosis and treatment since 2000. However, there is concern that the COVID-19 pandemic will reverse the recent progress in reducing the TB global burden due to TB services disruption leading to delayed diagnosis, including active case finding and contact tracing, as well as treatment interruption [1].

Radiology plays a crucial role in the diagnosis and monitoring of pulmonary tuberculosis (PTB). In this review we aim to update radiologists on four main topics to improve the understanding and diagnostic value of imaging in PTB. We will present the old, well-established findings in PTB, the new concepts in active PTB with special focus on immune status, the borrowed appearances from other disease in which the signs were initially described, and finally the imaging findings which commonly correlate with sputum smear positivity.

### Something old, something new

Tuberculosis is classified as primary if the onset of clinical disease falls within 1 year of the initial infection with *Mycobacterium tuberculosis*. Post-primary includes reactivation if the disease onset is more than 1 year after the initial exposure [2, 3], and reinfection with a different strain (especially in endemic areas) [4, 5]. Primary TB is most common in infants and children, with the highest prevalence under 5 years of age [6]. The prevalence of primary TB in adults is increasing, accounting for up to 34% of all adult cases of TB [6], particularly in developed countries [7]. Post-primary TB occurs in patients previously sensitised to *M. tuberculosis* and is considered a disease of adolescence and adulthood. Approximately 1 in 10 people with primary PTB present clinically. If untreated, approximately 1 in 10 cases reactivate, predominantly in a state of immunodeficiency [8].

Primary TB and post-primary TB are thought to be two distinct entities on the basis of clinical, pathologic and imaging findings [6]. Primary TB manifests radiologically as four main entities: dense, homogeneous consolidation with lower and middle lobes predominance, lymphadenopathy, miliary disease and pleural effusion [5]. Classical CT findings in post-primary PTB include centrilobular nodules, “tree-in-bud” sign, consolidation, ground-glass opacities, cavitation, bronchial wall thickening, miliary nodules, an isolated pulmonary nodule, parenchymal bands and interlobular septal thickening [8, 9].

The pathophysiology of primary TB has been described by Buzan et al. [10]. The disease is contracted through inhalation of 2–10 μm droplets laden with bacilli from an infected host. The mycobacteria reach the pulmonary alveoli preferentially in the best ventilated areas of the lungs, where they invade and replicate within alveolar macrophages. Granulomas develop after a few weeks, which can later progress to larger tuberculomas. Four to ten weeks after initial infection, delayed hypersensitivity manifests leading to a positive tuberculin reaction. Subsequently, caseous necrosis develops in the pulmonary focus—known as the Ghon focus—and/or in the involved lymph nodes. The primary focus and the involved lymph nodes form the primary complex, called the Ranke or Ghon complex. Typically the Ghon focus undergoes healing resulting in a visible scar that may contain foci of calcification [7, 11], though in some cases it enlarges as disease progresses. Different factors such as number and virulence of the mycobacteria, natural and acquired resistance of the host, and hypersensitivity influence the extent of the primary infection [12]. Disease progression can manifest as: local progression (consolidation, cavitation), bronchogenic dissemination (centrilobular and tree-in-bud nodularity), haematogenous dissemination (miliary nodules) and lymphatic dissemination [13]. Corresponding histopathology features of active TB are presented in Additional file 1: Table S1.

Lower lobe disease, adenopathy, and pleural effusions are common in children but less common in adults. These findings therefore became known as atypical disease in adults, whilst the upper lobe disease observed in most adults became known as typical reactivation tuberculosis [2]. The term “atypical” might be misleading because certain patterns are in fact typical for the immunocompromised status [14]. Although most tuberculosis cases in immunocompromised individuals are related to reactivation of latent tuberculosis, the radiological and clinical manifestations more frequently resemble primary TB [4]. Upper lobe cavitary disease is usually seen in infected immunocompetent hosts, whereas immunocompromised patients often present with lower-lung disease, adenopathy and effusions [2]. In the paediatric population of active TB, an upper lobe predominant distribution was identified in 36–82% of cases [2, 15] which was associated with lymphadenopathy in up to 15% of cases [15]. Imaging appearances of active PTB are independent of the time since infection [16, 17] and thus cannot differentiate primary from post-primary TB [2, 17]. In fact, the advances in molecular epidemiology in the 1990s have led to the discovery that the radiographic appearances of TB depends on immune status and therefore the suggested terminology for TB infection is “active TB” [2].

**Fig. 1:**
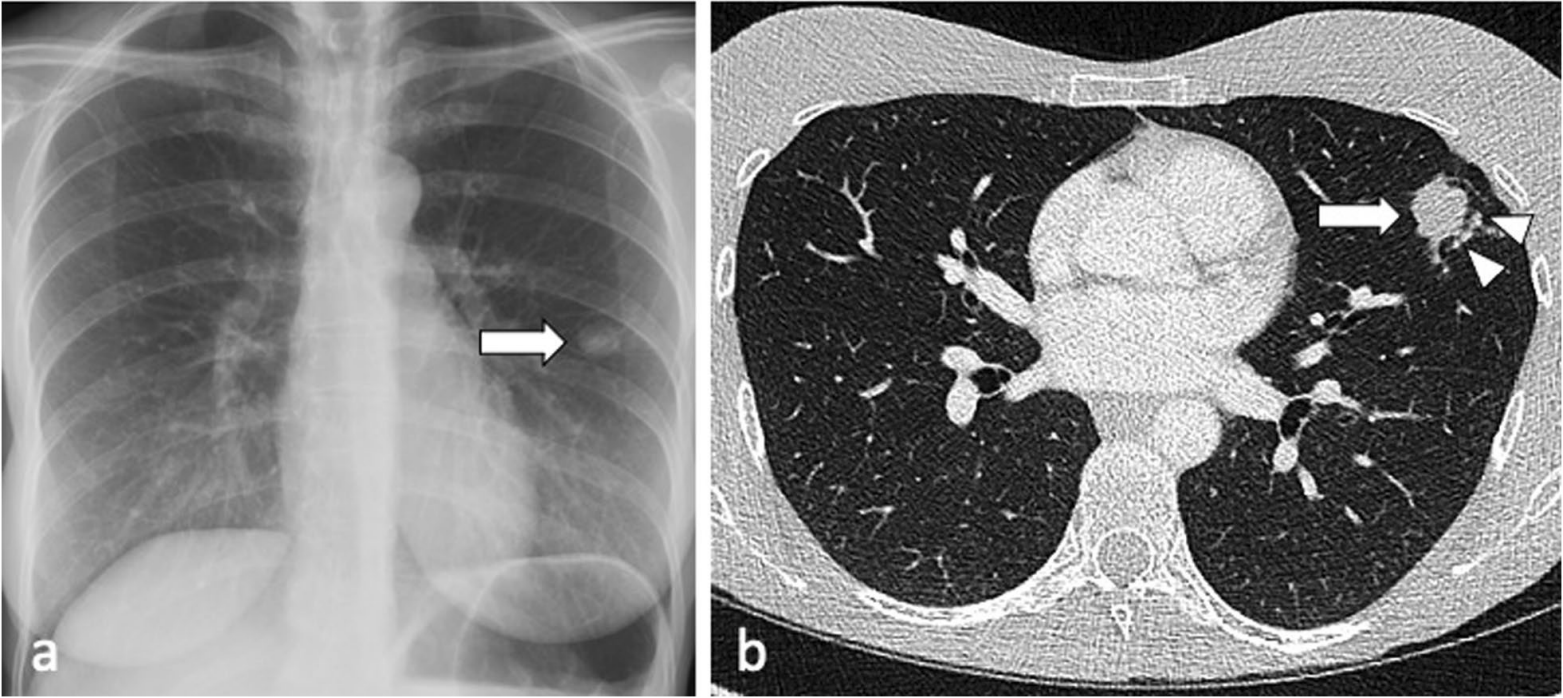
A 37-year-old female had a routine chest radiograph (**a**) which demonstrated a solitary pulmonary nodule in the left mid zone (arrow). CT (lung window, axial plane—(**b**) confirms the presence of a 27 mm nodule in the lingula (arrow) with adjacent tiny satellite nodules (arrowheads) but no lymphadenopathy. Sputum smears were negative but TB culture from bronchoalveolar lavage was positive for *M. Tuberculosis*

Tuberculoma represents a pulmonary nodule and may be the only abnormality seen on chest radiographs in approximately 5% of patients with active TB [6]. Satellite nodules around the tuberculoma, with typically smooth, sharply defined margins, may be present in up to 80% of cases. When solitary, active tuberculomas may be mistaken for malignancy [6] (Fig. 1).

**Fig. 2:**
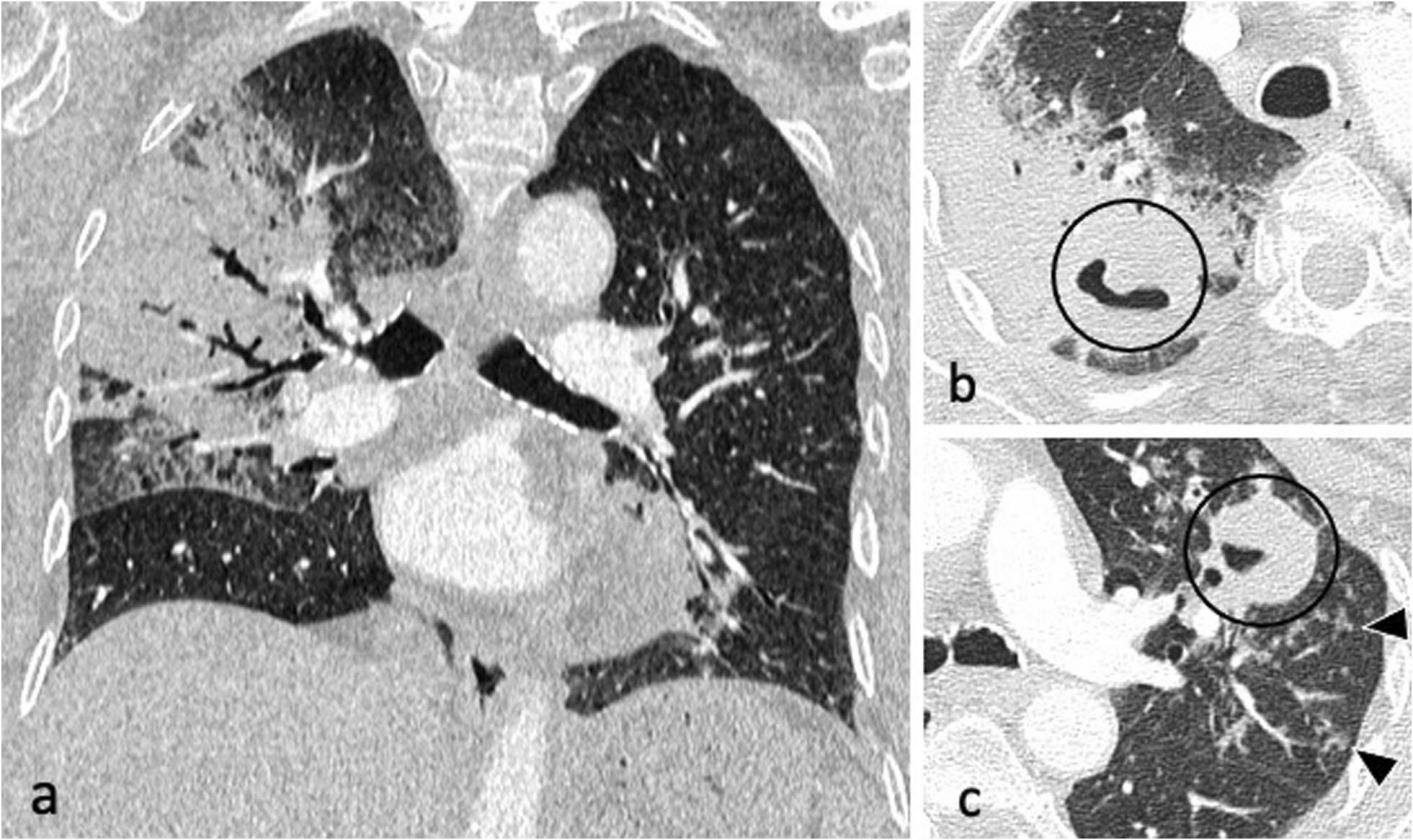
An 83-year-old male presented with pyrexia of unknown origin. CT (lung window) demonstrates extensive consolidation in the right upper lobe (coronal plane—**a**), bilateral thick-walled cavities (axial planes—**b**, **c**, circled) and centrilobular nodules with a tree-in-bud appearance (arrowheads)

Patchy, poorly defined areas of heterogeneous consolidation are among the earliest manifestations of active PTB. The distribution is primarily in the apical and posterior segments of the upper lobes and less frequent in the apical segments of the lower lobes, with commonly more than one pulmonary segment involved [5]. Tuberculous consolidation can be challenging to distinguish from bacterial pneumonia in absence of associated findings such as lymphadenopathy or cavitation and the lack of response to conventional antibiotics [6]. In their evolution, these regions liquefy and form cavities by draining through the tracheobronchial tree [10, 18, 19]. Cavitation affects about 50% of patients. The cavities are usually multiple and typically have thick, irregular walls, which become smooth and thin with successful treatment [10]. Cavities may demonstrate air-fluid levels which can also indicate superinfection [5] (Fig. 2), (Additional file 1: Fig. S1).

**Fig. 3:**
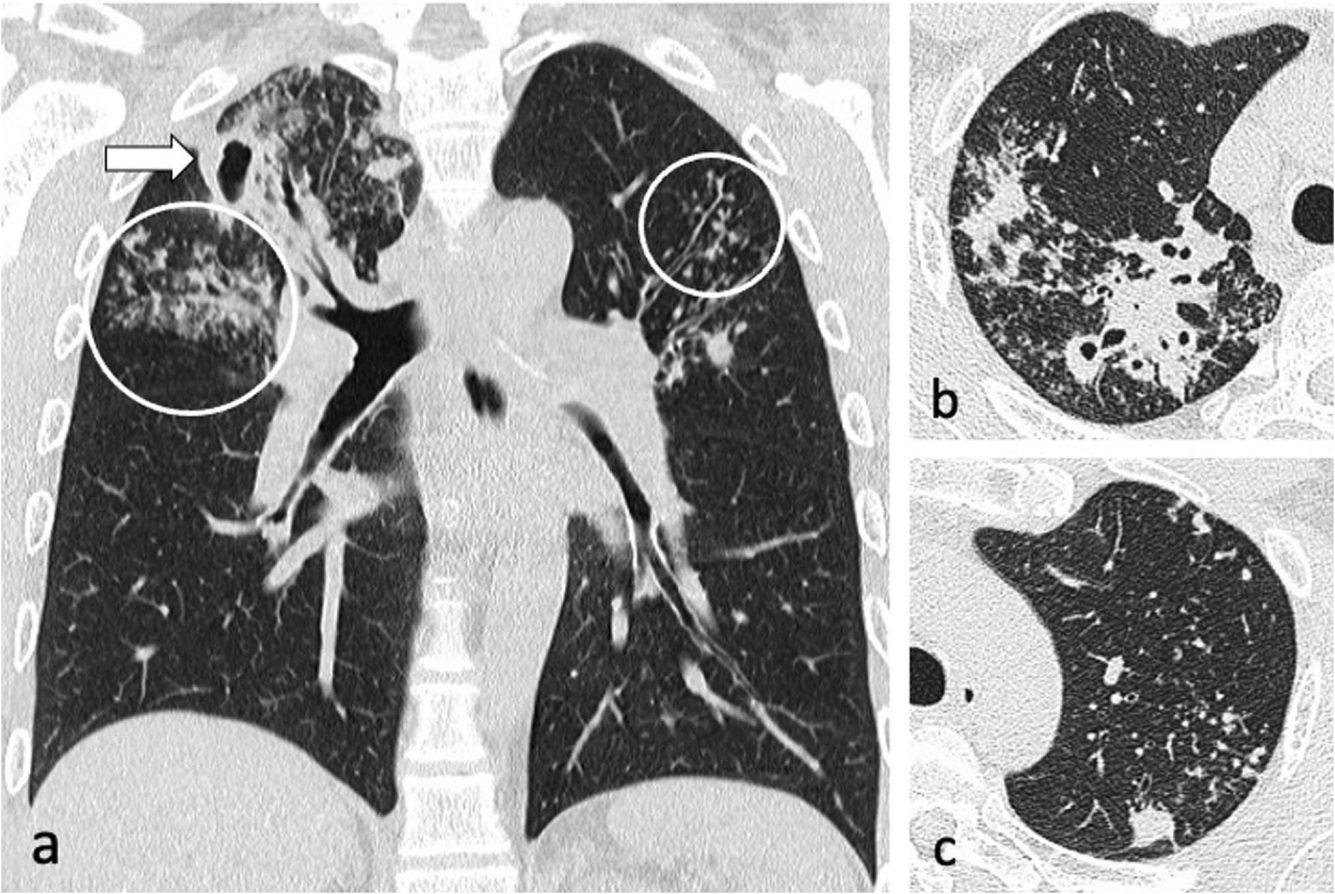
A 41-year-old male presented with chronic cough, weight loss and fevers. CT (lung window) demonstrates bilateral areas of centrilobular and tree-in-bud nodules (coronal plane—**a**, circled; axial plane—**c**) and consolidation in the right upper lobe (axial plane—**b**). There are also cavitating lesions with thick walls (arrow, **a**)

Bronchogenic spread manifests as multiple, 2–4 mm centrilobular nodules and sharply marginated linear branching opacities, described as a “tree-in-bud” pattern [7] (Fig. 3). These have a tendency to coalesce in a segmental or lobar distribution, typically involving the lower lung zones and the peripheral areas of consolidation or cavities [10]. Micronodules are difficult to identify on standard chest radiography [20], therefore CT is the imaging technique of choice to reveal early bronchogenic spread [7]. The term “tree-in-bud” was first used to describe the characteristic appearance of the endobronchial spread of TB [19], however, it is not pathognomonic for active TB [4, 21]. Patchy areas of air trapping are also seen in some patients with tuberculous bronchiolitis [6].

**Fig. 4:**
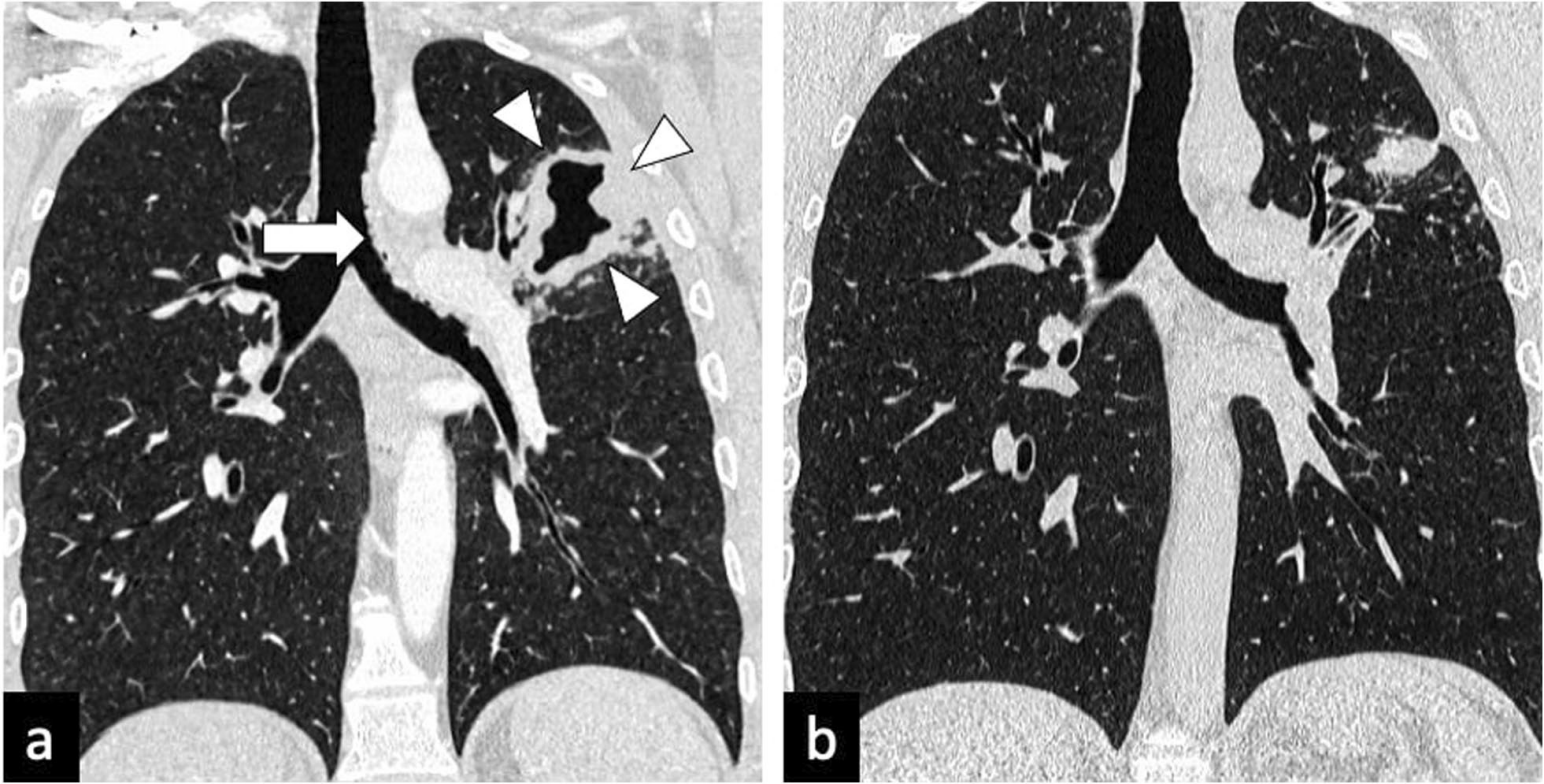
A 35-year-old male presented with a 4-week history of productive cough, anorexia and weight loss. Initial CT demonstrates tracheal and left main bronchus wall thickening with irregularity suggestive of ulcerations (lung window, coronal plane—**a**, arrow). There is also a thick-walled cavity with surrounding nodularity (arrowheads). The appearances significantly improved following TB treatment as demonstrated on a follow-up CT performed two months later (lung window, coronal plane—**b**)

Characteristic findings of central airway TB include irregular circumferential wall thickening with luminal narrowing. Isolated tracheal disease is rare with most patients presenting with distal trachea, carina and proximal main stem bronchi involvement [6, 11] (Fig. 4). The coexistence of tracheobronchial disease and lymphadenopathy is high in patients with active pulmonary TB [6]. In the pathophysiology of tracheobronchial TB, peribronchial lymphatic spread seems more common than endobronchial spread from infected sputum [6]. Bronchial stenosis occurs in 10–40% of patients with active tuberculosis [5, 22] and can lead to segmental or lobar atelectasis, lobar hyperinflation, mucoid impaction, and post-obstructive pneumonia [4].

**Fig. 5:**
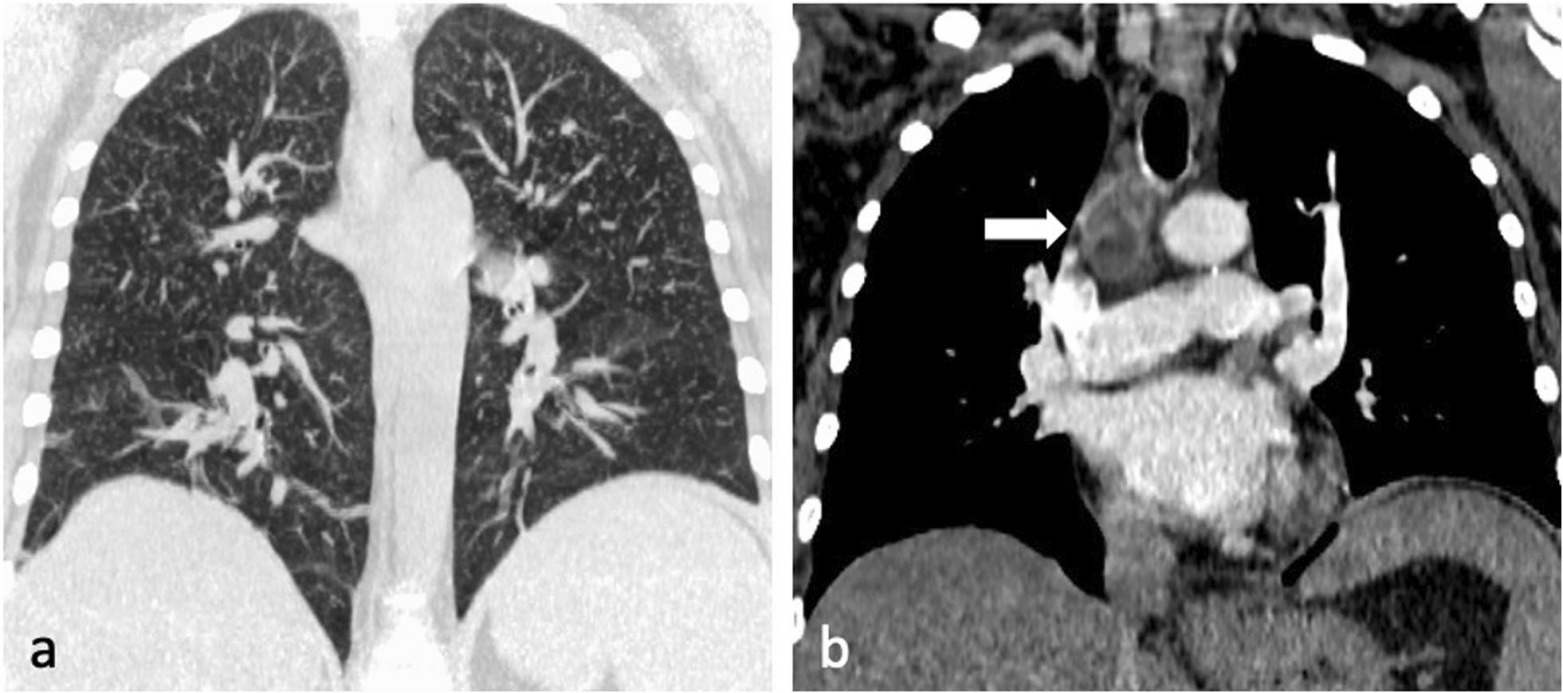
A 44-year-old female presented with symptoms of lethargy, loss of appetite, vomiting and fevers. She had been diagnosed with HIV two months prior but was not compliant with antiretroviral therapy. Her CD4 count at the time of presentation was 110 cells/mm^3^. CT demonstrates miliary nodules (lung window, coronal plane, maximum intensity projection—**a**) and an enlarged right paratracheal node with central low attenuation (mediastinal window, coronal plane—**b**, arrow) consistent with necrosis

Miliary TB describes haematogenous dissemination, resulting in randomly distributed nodules which have uniform size between 1 and 4 mm. They may have a slight lower lobe predominance, often associated with intra- and interlobular septal thickening [23], and may coalesce to form focal or diffuse consolidation [5]. Chest radiography is usually unremarkable at the onset of symptoms [5] and the nodules only become discernible after 4 weeks [8]. CT can reveal miliary disease before it becomes radiographically apparent [7]. Miliary TB may be seen in association with typical parenchymal changes or may be the only pulmonary abnormality [11]. Miliary disease has been reported to be associated with both childhood and immunocompromised adult infections [6, 7, 11] (Fig. 5a), manifesting within 6 months of initial exposure [5]. This pattern’s diffuse random distribution distinguishes it from the patchy centrilobular distribution of tree-in-bud [23]. Other organs with high blood flow, such as the liver, spleen, bone marrow, adrenals and kidneys, are also frequently affected [6].

**Fig. 6:**
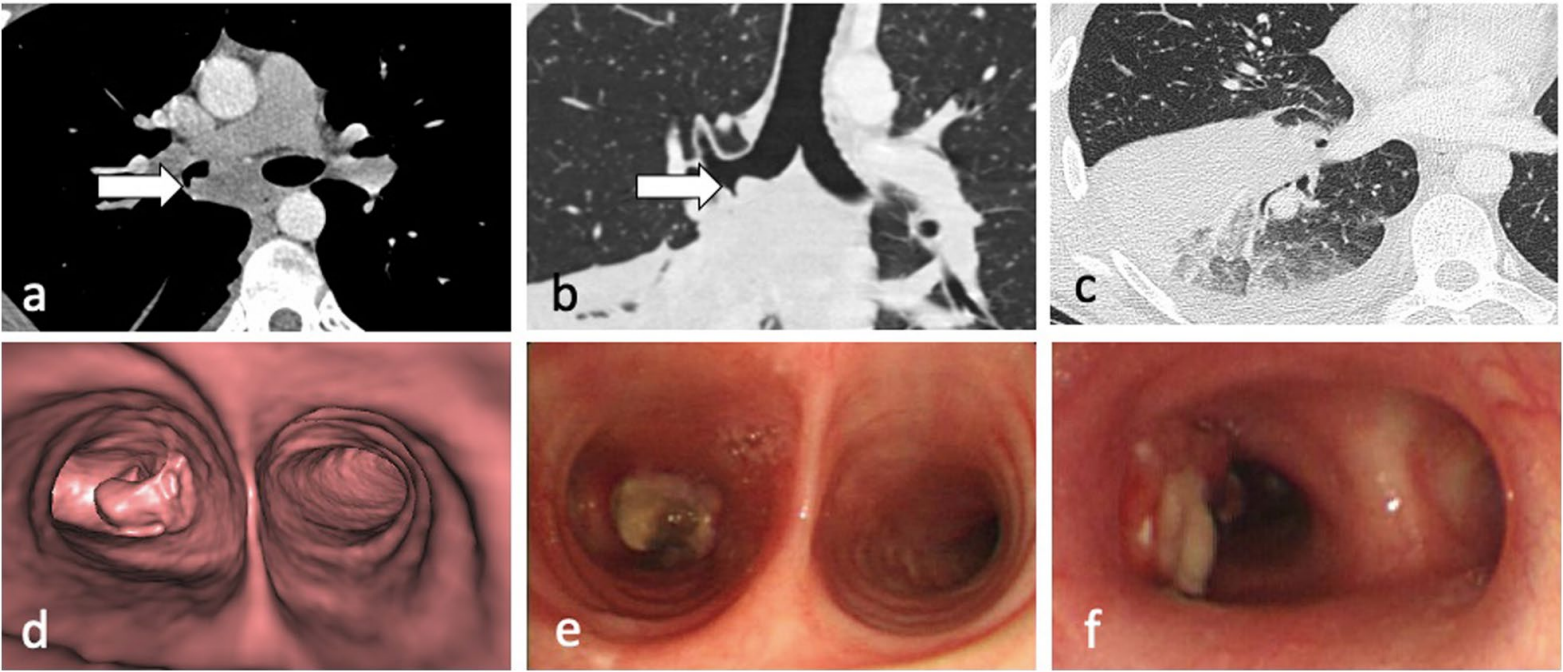
A 34-year-old male with Crohn’s disease presented with recurrent pyrexia and raised inflammatory markers. CT demonstrates a 29 mm subcarinal node with central low attenuation (mediastinal window, axial plane—**a**, arrow) invading into the right main bronchus (lung window, coronal plane—**b**, arrow). There is also right lower lobe consolidation and a small right pleural effusion (lung widow, axial plane—**c**). CT virtual bronchoscopy demonstrates the node protruding into the lumen of the right main bronchus (**d**) with corresponding bronchoscopy image (**e**). The patient underwent cryoprobe removal of the lymph node tissue successfully (bronchoscopy image—**f**)

Lymphadenopathy is typically unilateral, involving the right hilum and paratracheal region, but can be bilateral in about one-third of cases. It may represent the only radiographic finding or can be associated with parenchymal infiltrates on the same side as nodal enlargement, especially in the subpleural areas [10]. On CT, nodes greater than 2 cm in diameter may present with low-attenuation centres and peripheral rim enhancement [5, 6] (Fig. 5b). TB lymphadenopathy may be complicated by obstructive consolidation, obstructive atelectasis or hyperinflation secondary to compression of a bronchus by an adjacent enlarged node, perforation of a lymph node into a bronchus, typically at the level of a right lobar bronchus or bronchus intermedius [7, 24], (Fig. 6).

**Fig. 7:**
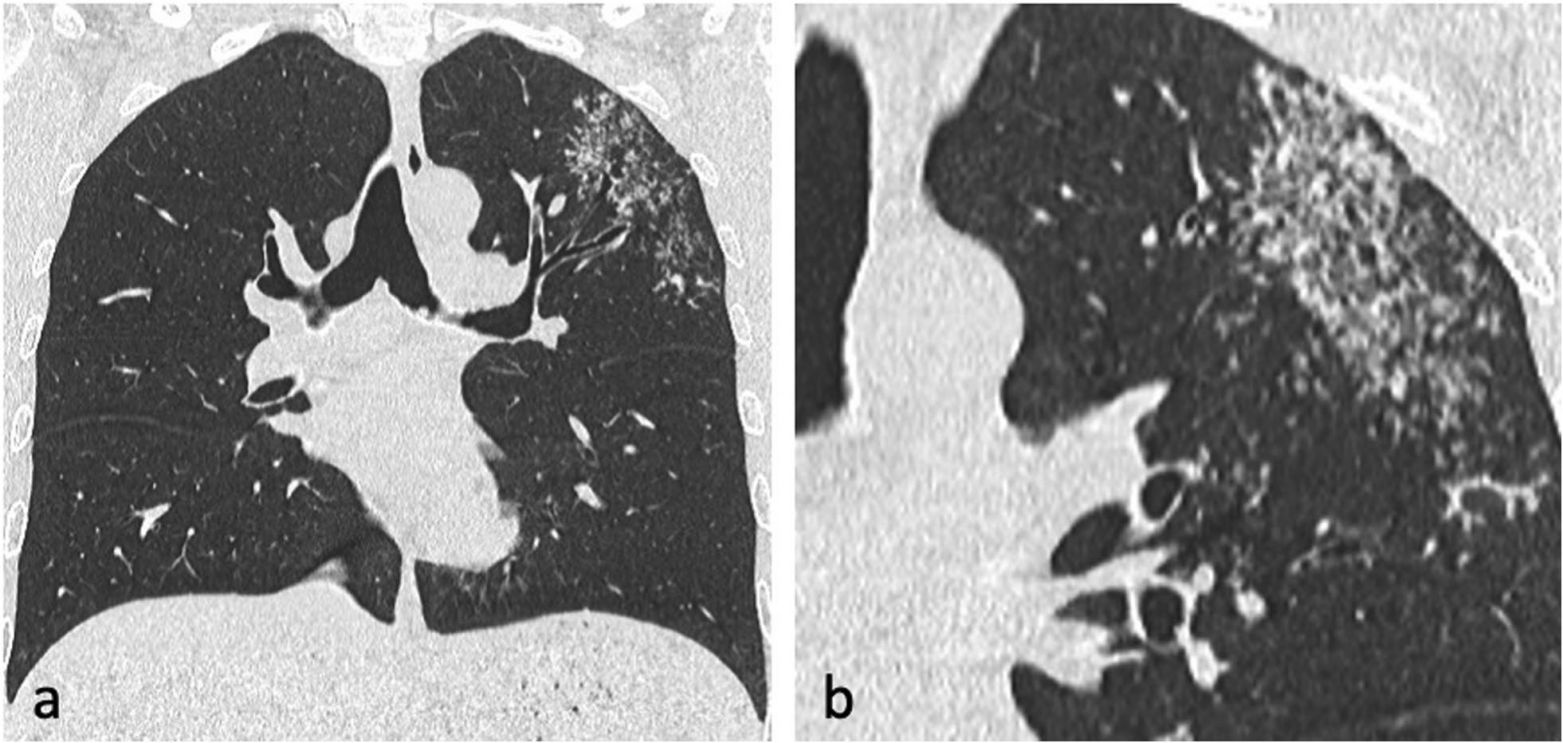
A 29-year-old male presented with cough and haemoptysis. CT (lung window, coronal planes—**a**, **b**) demonstrates a cluster of tiny nodules within the apicoposterior segment of the left upper lobe with perilymphatic distribution

The incidence of lymphatic dissemination may be higher than initially thought [13]. Perilymphatic micronodules were detected in up to 58% of active TB cases, with most of the nodules distributed along the bronchovascular bundles, but also along the interlobular septa and subpleural regions [13, 25]. A recent study failed to detect any evidence of lymphatic spread in the autopsied lungs of nine patients with advanced active PTB and the authors suggested that “clusters of micronodules” on CT represent aggregated tree-in-bud lesions [19]. However, PTB can be a lymphatic disease. Lymphatic spread is a major route of primary TB, and it could also explain the manifestation of TB resembling sarcoidosis and the negative sputum results despite extensive pulmonary lesions with micronodules on CT imaging [13, 25], (Fig. 7). Furthermore, lung biopsies in patients with early stage TB showing clusters of micronodules on CT and whose acid-fast bacilli (AFB) smear test and PCR assay were negative, demonstrated peribronchiolar granulomas [26]. Some granulomas were confined to the peribronchiolar interstitium with no caseation necrosis or invasion of the airway or alveolar space. Subpleural nodules have additional clinical significance, especially in young male patients, because they are thought to be responsible for the development of paradoxical response—an unusual expansion or new formation of a TB lesion within the immune reconstitution inflammatory syndrome during TB treatment [25]. This was described in 26% of patients with pleural TB [25]. Radiologically, lymphatic spread of TB is more common than bronchogenic spread in the lungs of patients with pleural TB [25].

**Fig. 8:**
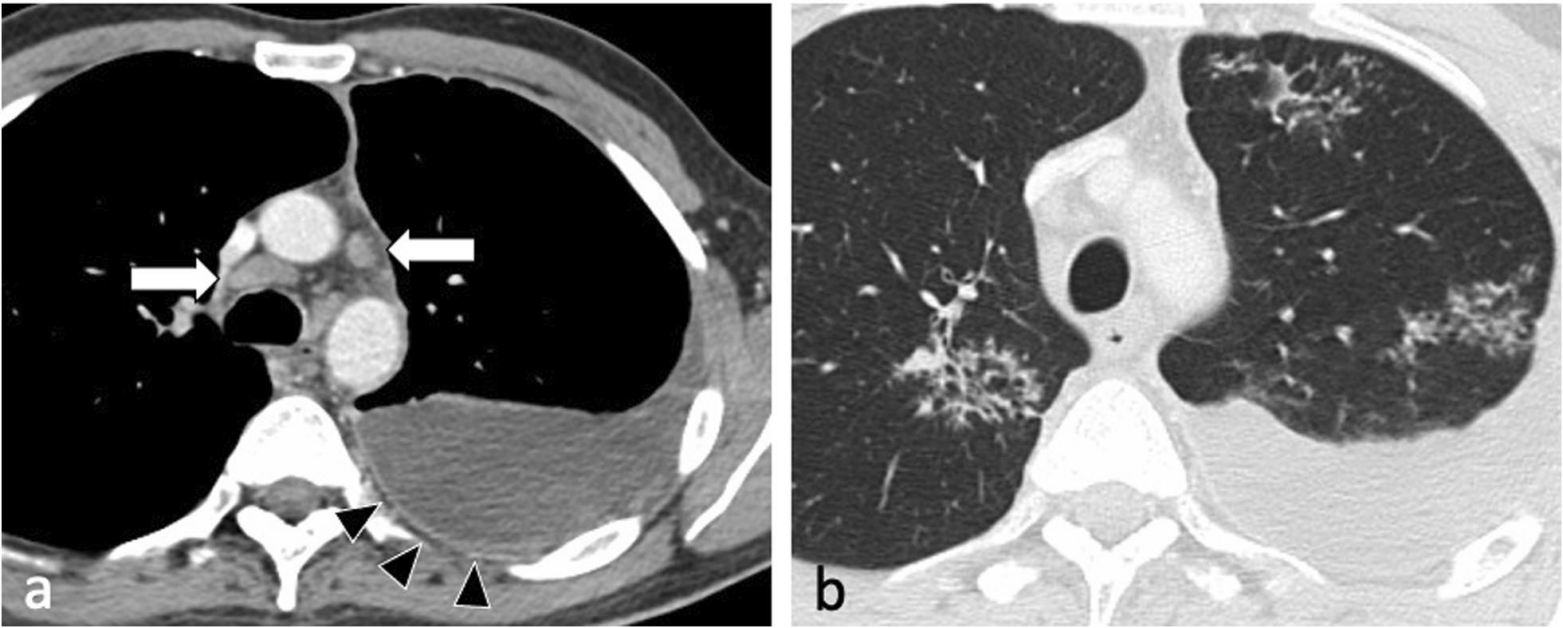
A 37-year-old male presented with intermittent pyrexia, sweats, rigors, reduced appetite and weight loss. Post-contrast CT shows a moderate volume left pleural effusion with pleural thickening and enhancement (mediastinal window, axial plane—**a**, arrowheads). There are also multifocal areas of perilymphatic nodularity (lung window, axial plane—**b**) and bilateral paratracheal and mediastinal lymphadenopathy (arrows, **a**). Pleural biopsy confirmed the presence of acid-fast bacilli

Pleural effusion may result from breakdown of subpleural foci and release of their contents into the pleural space directly or via pulmonary lymphatics followed by acute inflammation and exudation caused by delayed hypersensitivity reaction to tuberculous protein [4, 25]. However, frank pleural infection and isolation of *M. tuberculosis* from pleural fluid is uncommon. Tuberculous empyema is typically loculated and associated with pleural thickening and enhancement [4] (Fig. 8).

Known risk factors for development of active disease include conditions that are associated with defects in cell-mediated immunity: HIV infection, malignancy, malnutrition, prior gastrectomy or jejunoileal bypass, drug and alcohol abuse, end-stage renal disease, transplant recipients (Additional file 1: Fig. S2), diabetes mellitus, silicosis, corticosteroid or biological agents therapy, such as tumour necrosis factor α inhibitors for autoimmune disorders [4, 11]. These immunodeficient, high risk categories can present with mixed type patterns of active infection including anterobasal infiltrates, miliary nodules, hilar/mediastinal lymphadenopathy and exudative pleuritis, frequently combined with formation of cavities, as well as extrathoracic manifestations. This combination of findings may pose diagnostic challenges and delay treatment [7, 11]. For example, atypical location of TB in the lower zone can lead to a misdiagnosis of pneumonia, carcinoma, or lung abscess [27].

**Fig. 9:**
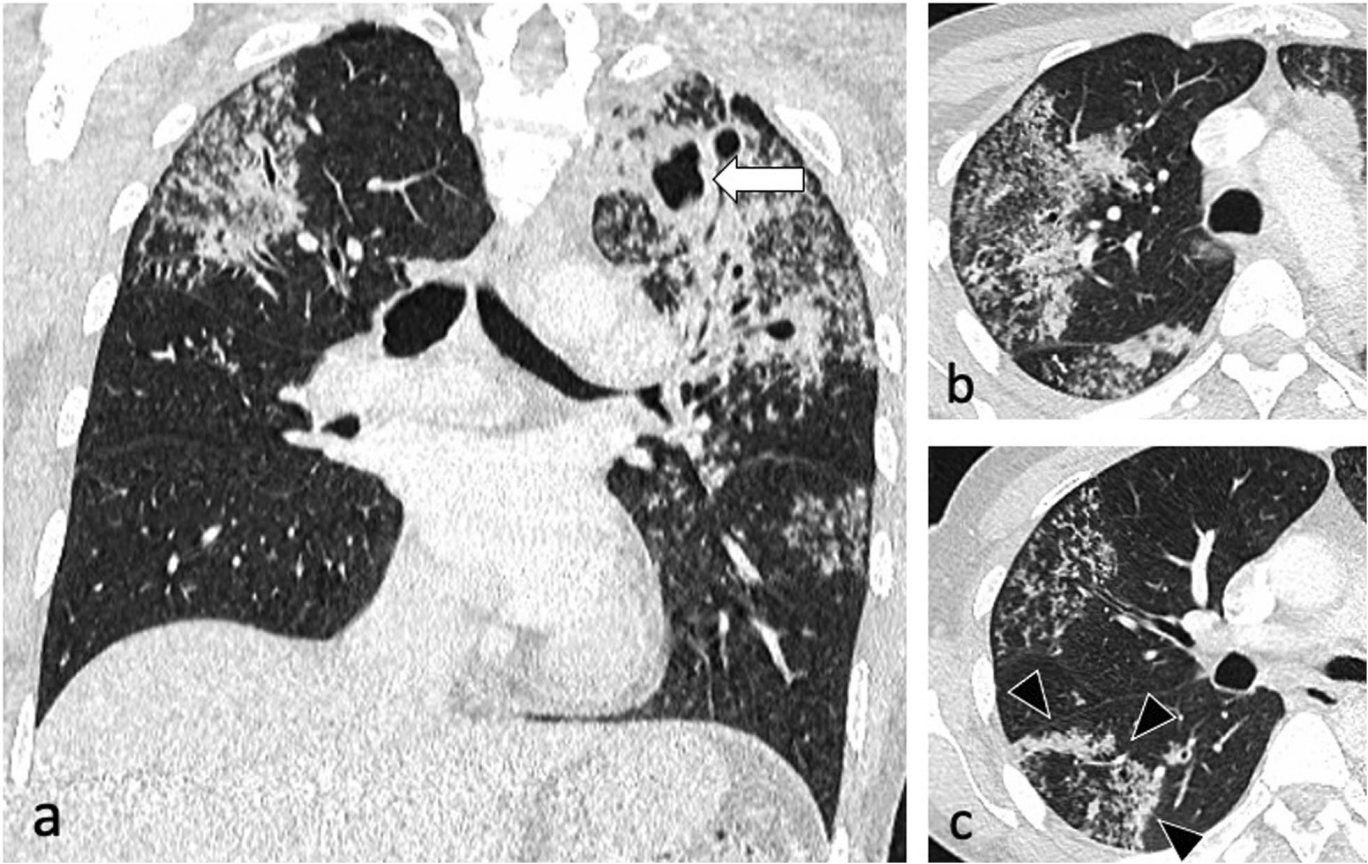
A 47-year-old female with a known diagnosis of HIV presented with weight loss, night sweats and worsening dyspnoea. Her CD4 count at presentation was 120 cells/mm^3^. CT (lung window) demonstrates thick-walled irregular cavities (coronal plane—**a**, arrow), extensive bilateral upper lobe centrilobular and tree-in-bud nodules and nodular reversed halo sign in the apical segment of the right lower lobe (axial plane—**c**, arrowhead)

**Fig. 10:**
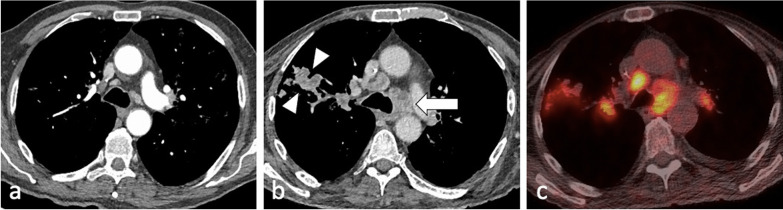
A 74-year-old male with HIV and a CD4 count of 10 cells/mm^3^ was started on antiretroviral therapy. He developed immune reconstitution inflammatory syndrome with worsening imaging findings in keeping with paradoxical reaction. Compared to the CT from three months prior (mediastinal window, axial plane—**a**), there are enlarged centrally necrotic precarinal lymph nodes (mediastinal window, axial plane—**b**, arrow) and necrotic nodules in the right upper lobe (arrowheads). These were avid on PET-CT (**c**)

HIV infection is the strongest known risk factor for developing active tuberculosis, with HIV-positive patients that have latent TB infection being 20–30 times more likely to develop active tuberculosis compared to HIV-negative patients [4]. The radiographic appearance of HIV-associated PTB is dependent on the level of immunodeficiency [7, 8, 11, 28] (Fig. 4 and 9). Patients with AIDS may present with extensive haematogenous dissemination following primary infection, and thus have a high risk of developing rapidly progressive primary TB during the first year after infection [7]. Because of deficient cell-mediated immunity, they are also prone to reactivation TB. A paradoxical clinical and radiographic worsening related to an increase in the CD4 counts and a decrease in the viral loads was observed within 60 days after the initiation of highly active antiretroviral therapy, known as immune reconstitution inflammatory syndrome [4, 6] (Fig. 10). Typical imaging findings include intrathoracic or cervical lymphadenopathy in approximately 70% of patients, new or increasing areas of consolidation and pleural effusions, as well as intraabdominal and neurologic manifestations [6].

A pattern of noncavitary consolidation, pleural effusion and lymphadenopathy is most common in patients with primary drug resistance, whereas cavitary disease is common in patients who acquired multidrug-resistant TB secondary to noncompliance with therapy [10, 29]. One review article describes an advanced pattern of extensive consolidation with or without lymphadenopathy in AIDS patients with extensively-drug-resistant PTB [12].

### Something borrowed—unusual patterns

Some radiological features of TB may mimic those of other diseases in which they were initially described. These “borrowed” signs do not represent well-known characteristics of TB and may be considered an unusual appearance of perilymphatic micronodules.

**Fig. 11:**
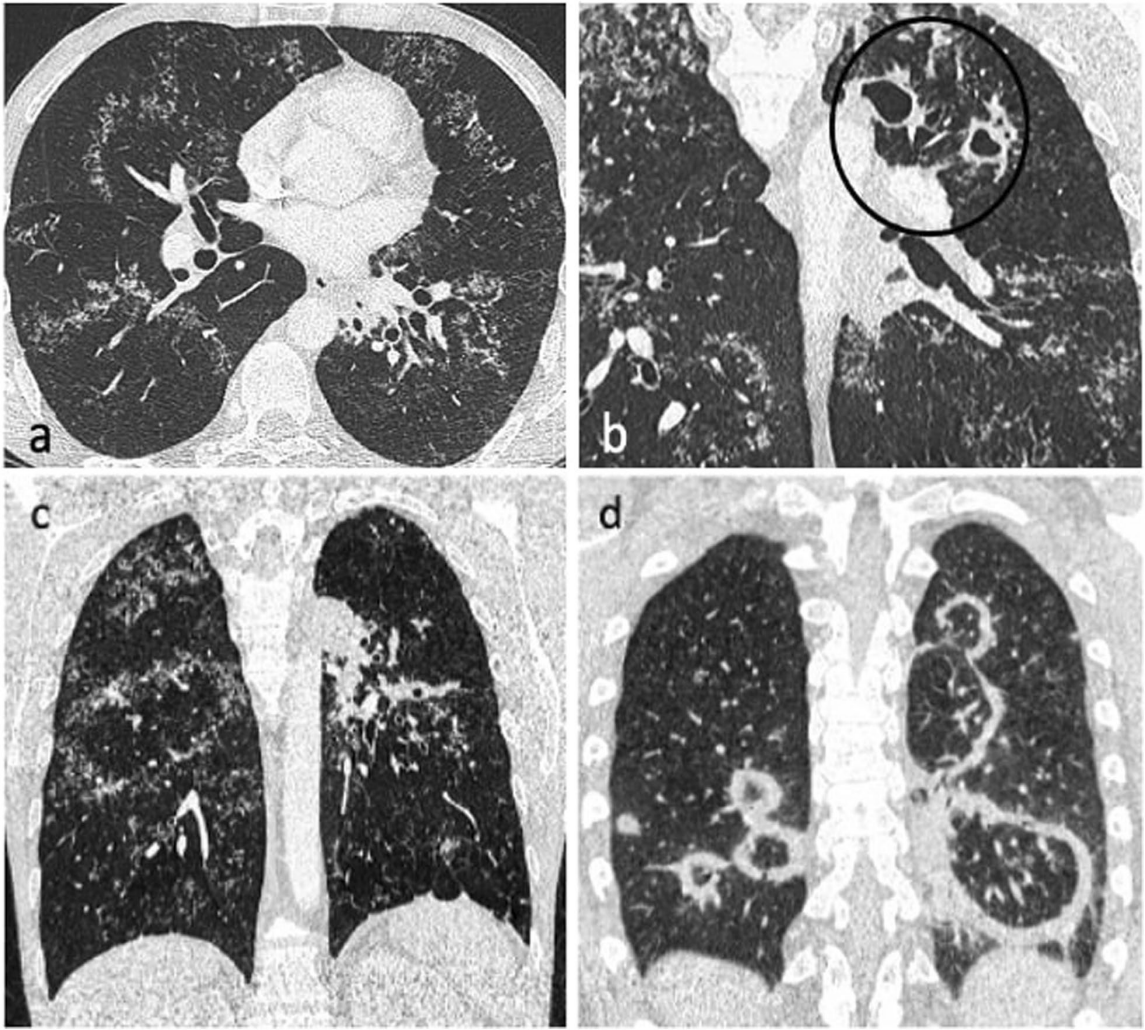
A 37-year-old male with active pulmonary TB presented with a 6-month history of weight loss and night sweats. CT (lung window) demonstrates nodular reversed halo sign in the right lower and middle lobe (axial plane—**a**, coronal plane—**c**), and cavitating lesions in the left upper lobe (coronal plane—**b**, circled). Image (**d**) is of a 25-year-old male with cryptogenic organising pneumonia who presented with cough and night sweats. CT (lung window, coronal plane) demonstrates multiple areas of rim consolidation reversed halo sign

The Fleischner Society defines the reversed-halo sign (RHS) as “a focal, rounded area of ground-glass opacity surrounded by a more or less complete ring of consolidation” seen on CT images [30]. In 1999, Zompatori et al. used the term “atoll sign” to describe a similar CT finding in a case of cryptogenic organising pneumonia (COP) [31]. COP is the most frequent cause of the RHS, but a wide spectrum of diseases can manifest with this sign. Recently, the presence of RHS has been described in patients with PTB [32]. Patients with TB present with a nodular pattern of RHS characterised by the presence of micronodules representing granulomas within the wall and inside the reversed halo [9, 33] (Fig. 9c and 11a, c). This morphological appearance of the ring and ground-glass component with presence of small nodules usually indicates active TB, rather than organising pneumonia [9] (Fig. 11). A recent study revealed an incidence of nodular RHS of 17% in a cohort of patient with active PTB [34]. The RHS in TB was described mainly in the right upper lobe with one or two such lesions being identified in most of the cases [9, 34]. Furthermore, RHS was associated with perilymphatic-predominant nodularity but not centrilobular-predominant nodularity in another recent study [13] (Fig. 11a).

The galaxy sign (GS) is a large parenchymal nodule arising from the coalescence of small nodules with many tiny surrounding satellite nodules (Additional file 1: Fig. 3). The cluster sign (CS) is also characterised by clusters of multiple small nodules but the nodules do not tend to coalesce (Fig. 6 and 7a). While initially defined in sarcoidosis [35, 36], these two signs were also described in TB [37, 38]. Heo et al. suggested the term “clusters of small nodules” instead of GS for better correlation with pathological morphology [39]. Both GS and CS seem to be post-primary in immunocompetent individuals and were described as associated with perilymphatic-predominant nodularity rather than centrilobular-predominant nodularity [13]. A recent study showed the time interval for minimal radiologic progression of these lesions was greater than 6 months and their extent increased with disease progression, frequently accompanied by a pattern of bronchogenic spread and consolidation [26].

### Something blue—patterns associated with positivity of AFB smears

Establishing diagnosis and activity of PTB is usually based on the detection of AFB in sputum smears or culture [10]. WHO defines definitive sputum smear-positive active PTB as: 1 sputum smear examination positive for AFB, plus a sputum culture positive for *M. tuberculosis*, or 2 or more initial sputum smear examinations positive for AFB [40]. The lipid-rich mycobacterial cell wall binds basic fuchsin dyes, and the staining is acid and alcohol resistant; therefore, these mycobacteria are termed AFB. The most commonly used acid-fast staining technique is Ziehl–Neelsen. The frequency of transmission from patients with presence of AFB on Ziehl–Neelsen stain is 22% higher than that from patients with negative smear [20, 41]. The sensitivity of three successive expectorated sputum smears for AFB ranges 68–72% in patients with culture-positive tuberculosis and is approximately 62% in HIV-positive patients [4]. Patients with sputum-negative PTB are difficult to diagnose and may be missed at all points of care. There are several radiological signs that have been shown to correlate with smear-positive or negative samples, and radiologists should be aware of those signs as they play an important role in guiding the need for isolation and empirical anti-tuberculous therapy, while awaiting culture confirmation.

There is a significant correlation between radiologic extent of disease and the degree of smear positivity. Different CT findings such as cavitation, ground glass opacities (GGO), consolidation, nodules and bronchial lesions are significantly associated with smear-positive PTB and increase in AFB smear grade [18, 42]. Multiple of such lesions in the upper lobes and multiple lobe involvement are associated with smear-positive PTB [43, 44].

Cavitation represents an independent predictive factor of smear-positive sputum results [41]. A significant relationship was reported between mycobacterial load and both volume and number of cavities [45]. The degree of smear positivity was further found to be significantly correlated with cavity wall thickness and distance from cavity to nearest airway [18]. The latter results from the fact that opening and discharging into airways is easier for central cavities than for peripheral cavities, which further explains smear negativity in some patients with peripheral cavities on CT [18]. Cavitation is also associated with a prolonged time required for smear to turn negative after 2 months of treatment [45].

If consolidation involves multiple segments and lobes, positive AFB-smear results are likely [41, 43]. This is an expected finding as caseous necrosis within the consolidation containing bacilli can drain through the airways. It was found that consolidation score differs significantly between smear-positive and smear-negative patients, while GGO score showed significant correlation with the degree of smear positivity [18].

**Fig. 12:**
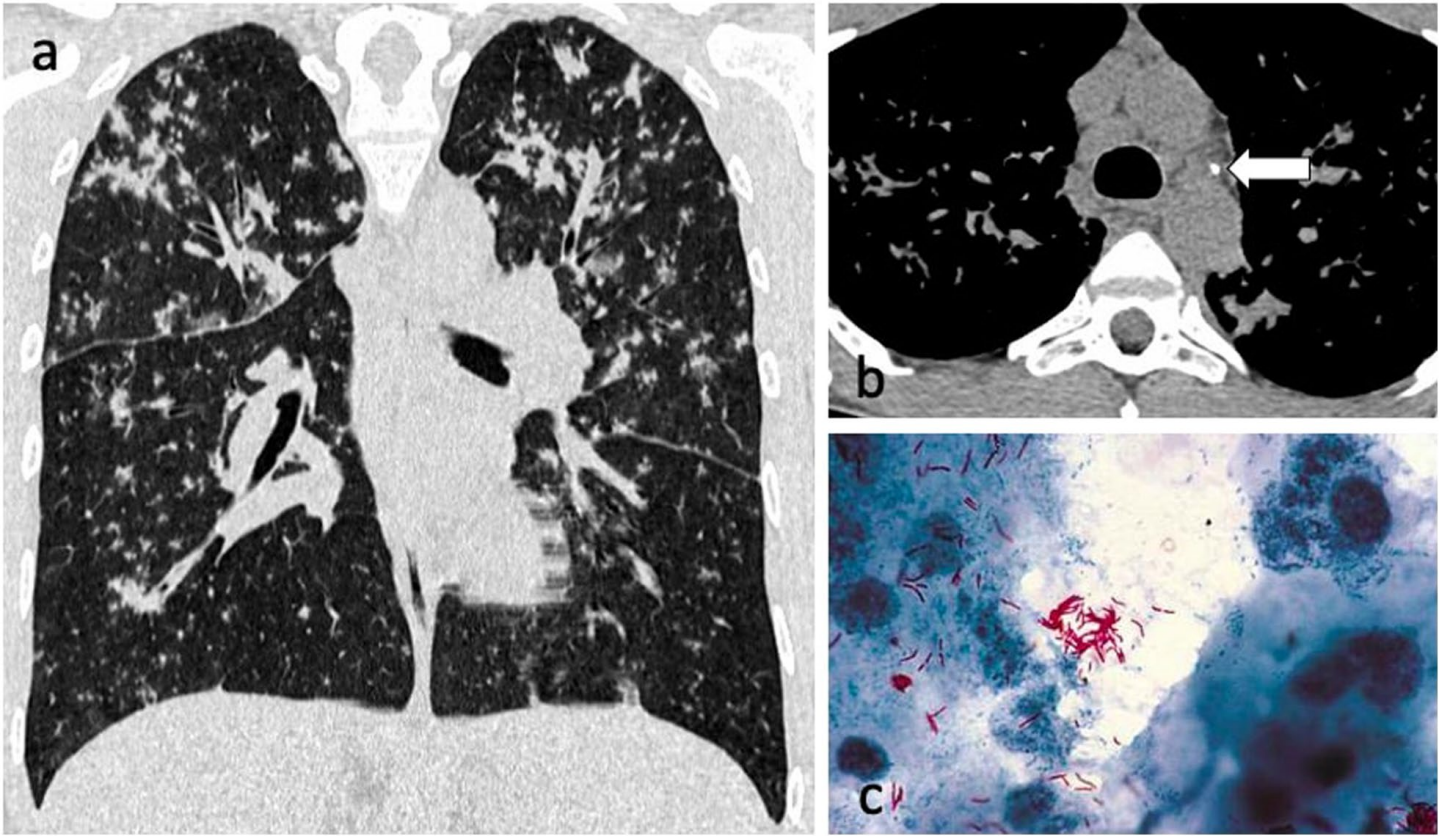
A 27-year-old male with Crohn’s disease on treatment with infliximab presented with a cough that was not resolving despite antibiotic therapy. CT demonstrates extensive centrilobular and tree-in-bud nodules with a mid-upper zone predominance (lung window, coronal plane—**a**). There are also small volume mediastinal and hilar lymph nodes (mediastinal window, axial plane—**b**). Ziehl–Neelsen stain was positive for AFB (**c**)

On the other hand, centrilobular nodules may not be associated with positive sputum smear results [41], due to a smaller affected volume containing less AFB-rich exudation and necrotic material, and a longer distance to the central airway compared to consolidation and cavitation [38]. This may explain the smear-negative cases presenting with tuberculomas such as the one in Fig. 1. A more recent study showed that the numbers of AFB present on sputum smears and the frequency of positive results for AFB in cases with centrilobular predominant nodularity were significantly higher than in cases with perilymphatic predominant nodularity [13] (Fig. 12).

Several studies and case reports have demonstrated that GS and CS were correlated with smear negative results [26, 40, 46–48]. Clusters of nodules was found to be a positive predictor of initially smear-negative active PTB [40]. Cases with clusters of micronodules as the predominant CT abnormality show significantly less sputum smear positivity than those without this CT predominant pattern [26]. Moreover, in a subgroup analysis which solely included subjects without cavity, AFB smear positivity was significantly lower in the clusters of micronodules-predominant cases. Similarly, RHS was reported in several smear-negative cases of PTB, probably due to a low rate of coexistent cavitation [9, 34]. The diagnosis of active PTB in these patients is usually based on culture positivity.

Further findings in smear-negative patients are consolidation in less than two lobes, absent cavitation or just in a single pulmonary lobe [44], and presence of pleural effusion [43, 49].

Similarly, in children with active TB there is an association between cavitation, tree-in-bud changes, and upper lobe infiltrates with smear positivity. Whilst lymphadenopathy and collapse were found to be associated with a negative smear [50]. These findings explain the false-negative sputum in up to 50% of AIDS patients with culture-proven TB [6, 51].

## Conclusion

We have presented the old, well-established findings in pulmonary TB, the new concepts in active pulmonary TB with special focus on immune status, the borrowed appearances from other disease which may pose diagnostic challenges, and the imaging findings which commonly correlate with sputum positivity. We hope this review will help improve understanding and diagnostic value of imaging in pulmonary TB and contribute to the continuous efforts led by WHO within the End TB strategy.

## Supplementary Information

**Additional file 1. Figure 1**. A 57-year-old male presented with shortness of breath, fevers, productive cough, haemoptysis, weight loss and night sweats. CT demonstrates multiple thick-walled cavities within areas of consolidation and centrilobular nodules (lung window, coronal plane – a, c, axial plane - b). There are also enlarged right paratracheal lymph nodes (mediastinal window, axial plane – d, arrow).** Figure 2**. A 39-year-old male with recent pancreas kidney transplant presented with fatigue, drenching night sweats and a mild cough. CT shows patchy consolidation in the left upper lobe with a large thick-walled cavity (lung window, coronal plane – a, axial plane - b) and an enlarged left lymph node with central low attenuation in the aortopulmonary window (mediastinal window, axial plane - c).** Figure 3**. A 26-year-old female with incidental lung changes on cardiac MRI. CT shows clustering of perilymphatic nodules in the left upper lobe giving rise to the “sarcoid galaxy sign” (lung window, axial plane -a). The coexistence of symmetrical mediastinal and hilar lymphadenopathy (mediastinal window, axial plane - b) helps in the differentiation of TB and sarcoidosis in the presence of sarcoid galaxy sign. Endobronchial lymph node biopsy revealed granulomatous inflammation but no* M. Tuberculosis* was identified on culture or PCR.** Table 1**. Computed tomography TB findings and underlying histopathology features.

## Data Availability

Not applicable.

## Abbreviations

AFB: Acid-fast bacilli
AIDS: Acquired immunodeficiency syndrome
COP: Cryptogenic organising pneumonia
CS: Cluster sign
CT: Computed tomography
GGO: Ground glass opacities
GS: Galaxy sign
HIV: Human immunodeficiency virus
PCR: Polymerase chain reaction
PTB: Pulmonary tuberculosis
RHS: Reversed-halo sign
TB: Tuberculosis
WHO: World Health Organisation
