# Supplementary material for: Active pulmonary tuberculosis: something old, something new, something borrowed, something blue
Source: Insights Imaging. 2022 Jan 9;13:3. doi: 10.1186/s13244-021-01138-8 (PMC8743064; doi:10.1186/s13244-021-01138-8)
Supplement: Supplementary file 1 — Additional file 1. Figure 1. A 57-year-old male presented with shortness of breath, fevers, productive cough, haemoptysis, weight loss and night sweats. CT demonstrates multiple thick-walled cavities within areas of consolidation and centrilobular nodules (lung window, coronal plane – a, c, axial plane - b). There are also enlarged right paratracheal lymph nodes (mediastinal window, axial plane – d, arrow). Figure 2. A 39-year-old male with recent pancreas kidney transplant presented with fatigue, drenching night sweats and a mild cough. CT shows patchy consolidation in the left upper lobe with a large thick-walled cavity (lung window, coronal plane – a, axial plane - b) and an enlarged left lymph node with central low attenuation in the aortopulmonary window (mediastinal window, axial plane - c). Figure 3. A 26-year-old female with incidental lung changes on cardiac MRI. CT shows clustering of perilymphatic nodules in the left upper lobe giving rise to the “sarcoid galaxy sign” (lung window, axial plane -a). The coexistence of symmetrical mediastinal and hilar lymphadenopathy (mediastinal window, axial plane - b) helps in the differentiation of TB and sarcoidosis in the presence of sarcoid galaxy sign. Endobronchial lymph node biopsy revealed granulomatous inflammation but no M. Tuberculosis was identified on culture or PCR. Table 1. Computed tomography TB findings and underlying histopathology features. [file 13244_2021_1138_MOESM1_ESM.docx]

**ELECTRONIC SUPPLEMENTARY MATERIAL**


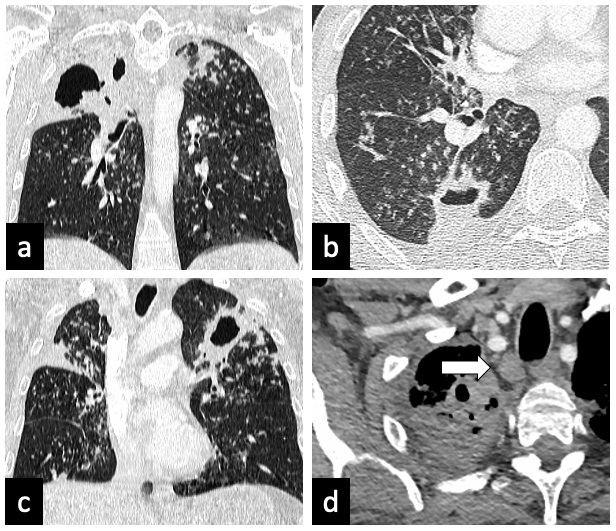


Figure 1: A 57-year-old male presented with shortness of breath, fevers, productive cough, haemoptysis, weight loss and night sweats. CT demonstrates multiple thick-walled cavities within areas of consolidation and centrilobular nodules (lung window, coronal plane – a, c, axial plane - b). There are also enlarged right paratracheal lymph nodes (mediastinal window, axial plane – d, arrow).


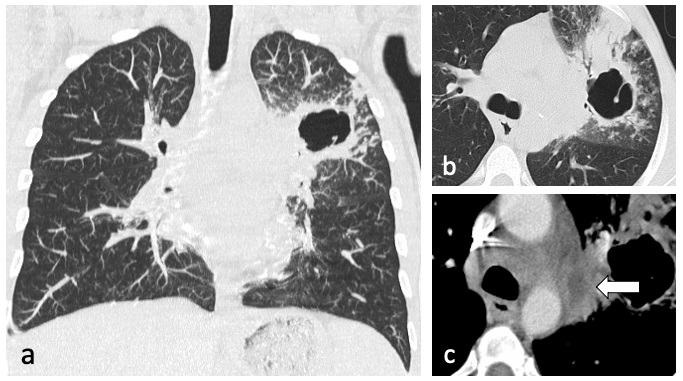


Figure 2: A 39-year-old male with recent pancreas kidney transplant presented with fatigue, drenching night sweats and a mild cough. CT shows patchy consolidation in the left upper lobe with a large thick-walled cavity (lung window, coronal plane – a, axial plane - b) and an enlarged left lymph node with central low attenuation in the aortopulmonary window (mediastinal window, axial plane - c).


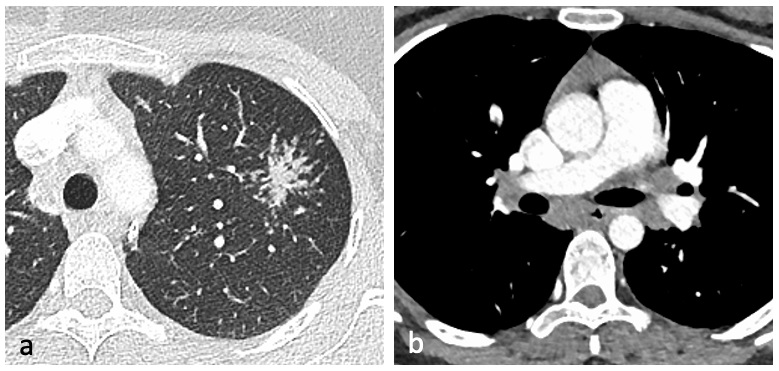


Figure 3: A 26-year-old female with incidental lung changes on cardiac MRI. CT shows clustering of perilymphatic nodules in the left upper lobe giving rise to the “sarcoid galaxy sign” (lung window, axial plane -a). The coexistence of symmetrical mediastinal and hilar lymphadenopathy (mediastinal window, axial plane - b) helps in the differentiation of TB and sarcoidosis in the presence of sarcoid galaxy sign. Endobronchial lymph node biopsy revealed granulomatous inflammation but no *M. Tuberculosis* was identified on culture or PCR.

Table 1. Computed tomography TB findings and underlying histopathology features

| **CT sign** | **Histopathology/cytology/biochemistry** |
| --- | --- |
| Lymphadenopathy | Central caseation necrosis and peripheral granulomatous inflammatory tissue [1, 2] |
| Consolidation | Caseation necrosis (coagulation necrosis characterised by disintegration of lipid-rich cells and conversion into a homogeneous structure without blood vessels) and peripheral nonspecific inflammation [3] |
| Tuberculoma | Central core of caseating necrosis with a surrounding wall of a granulomatous reaction containing Langerhans giant cells, epithelioid histiocytes, and lymphocytes [2] |
| Pleural effusions | Exudate with lymphocytic predominance; granuloma in the parietal pleura; elevated adenosine deaminase level [2] |
| Tree-in-bud | The terminal tufts represent endoluminal material (caseum, inflammatory cells and debris, fibrosis, and granuloma formation depending on chronicity) within the alveolar ducts, while the stalk represents affected bronchioles and last order bronchus within the secondary lobule [3, 4] |
| Perilymphatic nodules | Noncaseating necrotizing granulomas along the lymphatic vessels in the pulmonary interstitium (peribronchial, interlobular, subpleural), with no invasion of the airway or alveolar space and no fibrosis; larger nodules may represent caseating granulomas involving lung parenchyma [5–7] |

References:

1. Burrill J, Williams CJ, Bain G, et al (2007) Tuberculosis: a radiologic review. Radiogr Rev Publ Radiol Soc N Am Inc 27:1255–1273. https://doi.org/10.1148/rg.275065176

2. Restrepo CS, Katre R, Mumbower A (2016) Imaging Manifestations of Thoracic Tuberculosis. Radiol Clin North Am 54:453–473. https://doi.org/10.1016/j.rcl.2015.12.007

3. Im JG, Itoh H, Shim YS, et al (1993) Pulmonary tuberculosis: CT findings--early active disease and sequential change with antituberculous therapy. Radiology 186:653–660. https://doi.org/10.1148/radiology.186.3.8430169

4. Im J-G, Itoh H (2018) Tree-in-Bud Pattern of Pulmonary Tuberculosis on Thin-Section CT: Pathological Implications. Korean J Radiol 19:859–865. https://doi.org/10.3348/kjr.2018.19.5.859

5. Ko JM, Park HJ, Kim CH (2015) Clinicoradiologic evidence of pulmonary lymphatic spread in adult patients with tuberculosis. AJR Am J Roentgenol 204:38–43. https://doi.org/10.2214/AJR.14.12908

6. Kim J, Lee IJ, Kim JH (2017) CT findings of pulmonary tuberculosis and tuberculous pleurisy in diabetes mellitus patients. Diagn Interv Radiol Ank Turk 23:112–117. https://doi.org/10.5152/dir.2016.16157

7. Matsuoka S, Uchiyama K, Shima H, et al (2004) Relationship between CT findings of pulmonary tuberculosis and the number of acid-fast bacilli on sputum smears. Clin Imaging 28:119–123. https://doi.org/10.1016/S0899-7071(03)00148-7
